# Making Group Decisions within the Framework of a Probabilistic Hesitant Fuzzy Linear Regression Model

**DOI:** 10.3390/s22155736

**Published:** 2022-07-31

**Authors:** Ayesha Sultan, Wojciech Sałabun, Shahzad Faizi, Muhammad Ismail, Andrii Shekhovtsov

**Affiliations:** 1 Department of Statistics, Lahore Campus, COMSATS University Islamabad, Islamabad 45550, Pakistan; ayehsasultan@gmail.com (A.S.); muhammadismail@cuilahore.edu.pk (M.I.); 2 Research Team on Intelligent Decision Support Systems, Department of Artificial Intelligence and Applied Mathematics, Faculty of Computer Science and Information Technology, West Pomeranian University of Technology in Szczecin, ul. Zołnierska 49, 71-210 Szczecin, Poland; andrii-shekhovtsov@zut.edu.pl; 3 National Institute of Telecommunications, Szachowa 1, 04-894 Warsaw, Poland; 4 Department of Mathematics, Virtual University of Pakistan, Lahore 54000, Pakistan; shahzadfaizi@vu.edu.pk

**Keywords:** PHFS, FLRM, PHFLRM, peters model, MCDM

## Abstract

A fuzzy set extension known as the hesitant fuzzy set (HFS) has increased in popularity for decision making in recent years, especially when experts have had trouble evaluating several alternatives by employing a single value for assessment when working in a fuzzy environment. However, it has a significant problem in its uses, i.e., considerable data loss. The probabilistic hesitant fuzzy set (PHFS) has been proposed to improve the HFS. It provides probability values to the HFS and has the ability to retain more information than the HFS. Previously, fuzzy regression models such as the fuzzy linear regression model (FLRM) and hesitant fuzzy linear regression model were used for decision making; however, these models do not provide information about the distribution. To address this issue, we proposed a probabilistic hesitant fuzzy linear regression model (PHFLRM) that incorporates distribution information to account for multi-criteria decision-making (MCDM) problems. The PHFLRM observes the input–output (IPOP) variables as probabilistic hesitant fuzzy elements (PHFEs) and uses a linear programming model (LPM) to estimate the parameters. A case study is used to illustrate the proposed methodology. Additionally, an MCDM technique called the technique for order preference by similarity to ideal solution (TOPSIS) is employed to compare the PHFLRM findings with those obtained using TOPSIS. Lastly, Spearman’s rank correlation test assesses the statistical significance of two rankings sets.

## 1. Introduction

Statistical regression analysis is a valuable tool for determining the functional relationship between an output variable (the dependent variable) and the input variables (the independent variables). In statistical regression analysis, the relationship between IPOP variables is determined using precise data and precise relationships. However, when a phenomenon is imprecise, when there is vague variability rather than stochastic variability, and when the underlying regression model distributional assumptions are violated or cannot be tested (e.g., due to small sample size), it is more reasonable to assume a fuzzy relationship rather than a crisp relationship. Several researchers have modified and extended notions of statistical regression analysis to overcome these limitations using the fuzzy set theory (FST). Firstly, Tanaka et al. [1] introduced fuzzy regression analysis employing LPM. Further, Tanaka [2] introduced fuzzy intervals, Celmin [3], and Diamond [4] introduced fuzzy least-square models. Tanaka’s model was very sensitive to outliers, and then Peters [5] generalized Tanaka’s approach [1] where output values no longer fall within or outside the interval but rather belong to a certain degree of membership. Wang and Tsaur [6] presented a variable selection approach for a FLRM with crisp input and fuzzy output based on two criteria: the minimal total sum of vagueness and the minimal total sum of squares in estimation. Hong et al. [7] used fuzzy arithmetic operations to evaluate FLRMs based on Tanaka’s approach [1], where both IPOP data are fuzzy numbers (FNs). The solutions are derived using a generalized linear algorithm. Sakawa [7] modified FLRM by introducing two-phase construction of a linear regression model incorporating least-square estimation and LPM in different phases. Tanaka and Lee [8] used the proposed identification approach to perform exponential possibility regression analysis, resulting in a smaller region of possibility distribution that considered all possible sets of IPOP linear systems. To make LPMs more predictable and reduce computational effort, Modarres et al. [9] developed three FLRMs: risk seeking, risk neutral, and risk averse. They also developed a mathematical programming model to estimate FLRM parameters from crisp input and fuzzy output data. Parvathi et al. [10] introduced intuitionistic FLRM by incorporating an extension of FST called an intuitionistic fuzzy set into FLRM, where the parameters are symmetric triangular intuitionistic FNs. The parameters of an intuitionistic FLRM are estimated using an LPM that minimizes the total fuzziness of intuitionistic FLRM, which is associated with the width of intuitionistic fuzzy parameters. Sultan et al. [11] developed a fuzzy regression model employing HFS to solve a decision-making problem, in which IPOP variables are observed as hesitant fuzzy elements.

Nonlinear programming is used when the constraints or the objective function are nonlinear. To account for the nonlinear situation, Bárdossy [12]—considering regression models for FNs and the nonlinear problem—developed a generalized mathematical programming model. When the relationship between IPOP variables is intricate and nonlinear, determining the number of input variables for the model selection and the number of powers for input variables is challenging. Fuzzy regression analysis has also been studied from the perspective of the least-square approach, where the variability between the predicted fuzzy values and the actual fuzzy data is minimized for different distance measurements between two FNs. The fuzzy least-square method was initially proposed by Celmin [3] and Diamond [4] simultaneously, who estimated the fuzzy model parameters by minimizing the sum of squared error of the output variable.

The FST, introduced by Zadeh [13], is an excellent tool for describing ambiguous/vague information. The FST and their generalizations are powerful tools used in different fields [14,15]. The FST effectively employs membership functions and fuzzy numbers to deal with uncertainty in decision-making problems. It also has limitations when dealing with imprecise and vague data; as a result, the FST has been developed in a number of different directions, including the type-2 FSs [16], the hesitant FSs [17], probabilistic hesitant FSs [18], and the intuitionistic FSs [19] etc. Recently, the study of decision-making problems with the use of hesitant fuzzy information has become a significant focus of research, such as that of Liu et al. [20], who introduced a correlation coefficient approach to determine the strength of association between HFSs, which can be used to evaluate whether they are negatively or positively associated. Zeng et al. [21] introduced the weighted dual HFS, along with a few fundamental mathematical operations for weighted dual hesitant fuzzy elements, including union, intersection, multiplication, and complement; and Yan et al. [22] proposed a mathematical model for monitoring and evaluation bridge safety based on HFS. The HFS comprises a significant weakness in terms of data loss; to address this flaw, an extension of HFS called the PHFS has been proposed that enhances the HFS with probability and is capable of retaining more information than the HFS. Firstly, Zhu and Xu [18] developed a concept of PHFS, which incorporates distribution information into the HFS. Afterward, Zhang et al. [23] improved PHFE at first, then developed properties and aggregation operations for the modified PHFEs. In addition, Gao et al. [24] introduced a dynamic reference point technique using PHFS for emergency response that was based on probabilistic hesitant fuzzy information. Li and Wang [25] modified the QUALIFLEX approach to include probabilistic hesitant fuzzy environments and applied the suggested method to the selection of green suppliers; Wu et al. [26] developed a novel consensus-achieving approach for probabilistic hesitant fuzzy group decision making, and they implemented the suggested method to evaluate the strategic positions of energy transmission and distribution networks, and so on.

A single criterion is not enough in real-world decision-making problems, as they are often poorly structured and highly complex. Multi-Criteria Decision-Making (MCDM) methods solve complex problems and help to make the right decision. Finding the best alternative among the multiple alternatives is a challenging task. In decision-making problems, several techniques are used to assist DMs in ranking the alternatives, such as the Analytic Hierarchy Process [27], the Best Worth Method [28], EDAS (Evaluation on Distance from Average Solution) [29], and TOPSIS (a Technique for Order Performance by Similarity to Ideal Solution) [30]. The TOPSIS method is a well-known technique and considers the distances to both Positive Ideal Solution (PIS) and Negative Ideal Solution (NIS) simultaneously, and assigns a preference order based on their relative closeness and a combination of these distance measures. Recently, many papers have been devoted to developing new approaches, i.e., a new logarithm methodology of additive weights [31], FUCOM [32], COMET extensions [33,34], WASPAS method [35], SPOTIS [36,37], RAFSI [38], and an integrated SWOT–fuzzy PIPRECIA [39]. These methods are valuable and address the main challenges of MCDA techniques such as rank reversal paradox resistance or handling uncertainty. Sometimes, authors propose a new operators to support decision making [40,41,42].

The literature review shows how gradually the area of regression analysis has developed and how researchers continue to show increasing interest over time. We can see that most of the researchers’ attention has focused on the FLRM, a simple linear regression model developed using FST. Still, several extensions of the FST can be employed in the FLRM for complex problems. The PHFS works in a hesitant environment so that a researcher not only collects information in a HFS, but also finds its probability values for each HFE, which are referred to as PHFEs. Motivated by PHFS, a fuzzy regression model developed by Peters [5] has been extended using probabilistic information in a hesitant environment called PHFLRM, where IPOP variables are observed as PHFEs. We introduce the concept of PHFLRM such that the model’s coefficients are STFNs. Consequently, the PHFLRM incorporates these PHFEs into the fuzzy regression analysis and uses the LPM to estimate the PHFLRM parameters. Furthermore, alternatives are ranked according to the residual values of the proposed PHFLRM. The proposed approach is evaluated by comparing the results of PHFLRM to those of TOPSIS, which is the most popular MCDM technique. Previously, fuzzy regression models such as the FLRM [43] and HFLRM were used for decision-making; however, these models do not give distribution information. The novelty of our proposed model PHFLRM [11] is that it incorporates distribution information to account for multi-criteria decision-making (MCDM) problems.

This study is organized as follows: In Section 2, some basic definitions and terminologies are discussed. In Section 3, we establish the idea of PHFLRM. Section 4 includes an algorithm of the proposed approach PHFLRM. Section 5 presents an application example of the purposed approach, and a comparative study of the PHFLRM with TOSPSIS methods is discussed. This study concludes in Section 6.

## 2. Preliminaries

This section discusses basic definitions and terminologies to help readers understand the proposed approach. It is generally tough to reach a final conclusion, because people are usually hesitant when making decisions. Torra [17] developed the following definition of HFS in consideration of this problem:

**Definition** **1** ([17])**.** *For a fixed set Z, a HFS on Z is a function that, when applied to Z, returns a subset of values that fall within the interval [0,1]. Mathematically, it is defined as:*

$$E=\left\{\langle z,h_{E}\left(z\right)\rangle ,\;z\in Z\right\}$$

*where $h_{E}\left(z\right)$ denotes the possible hesitant membership degrees of z∈Z to set E, and it is called the hesitant fuzzy element.*

The PHFS proposed by Zhu and Xu [18] is an enhanced form of HFS that not only addresses the situation in which decision makers are uncertain as to which of several assessment values best represents their perspective, but also assigns varying probabilities to the assessed values. Mathematically, it is defined as:

**Definition** **2** ([18])**.** *Let Z be a reference set, then a PHFS on Z is defined as:*

$$E_{p}=\left\{z,h_{z}\left(\gamma_{l}|p_{l}\right),z\in Z\right\},$$

*where $h_{z}\left(\gamma_{l}|p_{l}\right)$ denotes the probabilistic degrees of memberships of the element z∈Z to set $E_{p}$. This is referred to as PHFEs, which can take several membership degrees $\gamma_{l}=\left(l=1,2,\cdots ,\#h_{z}\left(p\right)\right)$ with the probabilities $p_{l}=l=1,2,\cdots ,\#h_{z}\left(p\right))$ such that, $\underset{l=1}{\overset{\#h_{z}\left(p\right)}{\sum p_{l}=1}}$. For sake of convenience, we have assumed $h_{z}\left(\gamma_{l}|p_{l}\right)$ as $h_{z}\left(p\right)$ i.e., $h_{z}\left(p\right)$ = $h_{z}\left(\gamma_{l}|p_{l}\right)$.*

Sometimes, the probabilistic information for a PHFE is incomplete; in this situation, an estimate for the incomplete probabilistic information is used by averaging the available data.

**Definition** **3** ([23])**.** *If a PHFE $h_{z}\left(p\right)$ is given by$\;\sum_{l=1}^{\#h_{z}\left(p\right)}p_{l}<1$, then probabilities for the $h_{z}\left(p\right)$ are obtained as $p_{l}=\frac{p_{l}}{|\sum_{l=1}^{\#h_{z}\left(p\right)}{\;p}_{l}|},\;l=1,2,...,\#h_{z}\left(p\right).$*

 Some basic operations of PHFEs are defined as follows.

**Definition** **4.** *Let $h_{z}^{1}\left(p\right)$, ${h^{2}}_{z}\left(p\right)$ and $h_{z}\left(p\right)$ be three PHFEs; then, for any λ>0,*

*1.* *$\;{h^{1}}_{z}\left(p\right)⊕{h^{2}}_{z}\left(p\right)=\cup_{\gamma_{1_{l}}\in h_{z}^{1}\left(p\right),\gamma_{2_{k}}\in {h^{2}}_{z}\left(p\right)}\left\{\left[\gamma_{{}_{1_{l}}}+\gamma_{{}_{2_{k}}}-\gamma_{{}_{1_{l}}}\gamma_{{}_{2_{k}}}\right]\left(p_{1_{l}}p_{2_{k}}/\sum_{l=1}^{\#h_{z}^{1}\left(p\right)}p_{1_{l}}.\sum_{l=1}^{\#h_{z}^{2}\left(p\right)}p_{2_{k}}\right)\right\}$;*

*2.* *$\;h_{z}^{\lambda }\left(p\right)=\cup_{\gamma_{l}\in h_{z}\left(p\right)}\left\{\gamma_{l}^{\lambda }\left(p_{l}\right)\right\}$;*

*3.* *$\;\lambda h=\cup_{\gamma_{l}\in h_{z}\left(p\right)}\left\{\left[1-{\left(1-\gamma_{l}\right)}^{\lambda }\right]\left(p_{l}\right)\right\}$.*

**Definition** **5** ([23])**.** *Let $h_{z}\left(p\right)\;$ be a PHFE, the score function of $h_{z}\left(p\right)$ is defined as:*

$$S_{r}\left(h_{z}\left(p\right)\right)=\left(\sum_{l=1}^{\#h_{z}\left(p\right)}\gamma_{l}.p_{l}\right)/\sum_{l=1}^{\#h_{z}\left(p\right)}p_{l}$$

Let $h_{z}^{1}\left(p\right)\;$and $h_{z}^{2}\left(p\right)$ be two PHFEs, then

1. if $S_{r}\left(h_{z}^{1}\left(p\right)\right)>S_{r}\left(h_{z}^{2}\left(p\right)\right)$, then $h_{z}^{2}\left(p\right)<$$h_{z}^{1}\left(p\right)$;

2. if $S_{r}(h_{z}^{1}\left(p\right)=S_{r}\left(h_{z}^{2}\left(p\right)\right)$, then $h_{z}^{1}\left(p\right)=$$h_{z}^{2}\left(p\right)$.

**Definition** **6.** *Suppose $h_{z}^{1}\left(p\right)and\;h_{z}^{2}\left(p\right)$ are two PHFEs. Assuming $\#h_{1}\left(p\right)=\#h_{2}\left(p\right)$, the distance between $h_{z}^{1}\left(p\right)\;$and $h_{z}^{2}\left(p\right)$ is defined as*

$$D\left(h_{z}^{1}\left(p\right),h_{z}^{2}\left(p\right)\right)=\sqrt{\overset{\#h_{z}^{1}\left(p\right)=\#h_{z}^{2}\left(p\right)|}{\sum_{l=1}}{\left(\left(\gamma_{1_{l}}\right]\left(p_{l}\right)-\gamma_{2_{l}}\left(p_{l}\right)\right)}^{2}}$$

The distance measure $D(h_{z}^{1}\left(p\right),h_{z}^{2}\left(p\right))$ satisfies the following properties:

1. $D(h_{z}^{1}\left(p\right),h_{z}^{2}\left(p\right))<1;$

2.$D(h_{z}^{1}\left(p\right),h_{z}^{2}\left(p\right))=0$ if and only if $h_{z}^{1}\left(p\right)=h_{z}^{2}\left(p\right).$

3. $D\left(h_{z}^{1}\left(p\right),h_{z}^{2}\left(p\right)\right)=D\left(h_{z}^{2}\left(p\right),h_{z}^{1}\left(p\right)\right)$

## 3. Probabilistic Hesitant Fuzzy Linear Regression Model

In this section, we discuss our purposed methodology about PHFLRM from a statistical perspective using hesitant fuzzy information.

Initially, the FLRM was introduced by Tanaka et al. [1]. It is defined as:

$$\;\;\;{\hat{Y}}_{i}={\tilde{A}}_{0}+{\tilde{A}}_{1}X_{i1}+{\tilde{A}}_{2}X_{i2}+{\tilde{A}}_{3}X_{i3}+\cdots +{\tilde{A}}_{N}X_{iN},$$

where the parameters ${\tilde{A}}_{j}=\left(\alpha_{j},c_{j}\right)$ are symmetrical TFNs, $\alpha_{j}$ is the centre, and $c_{j}$ is the spread of the symmetrical TFNs. The FLRM minimizes the spread of the symmetrical TFNs in the following way [44]:

$$\min\overset{N}{\sum_{j=0}}\left(c_{j}\overset{M}{\sum_{i=0}}\left|x_{ij}\right|\right)$$

with following constraints
$y_{i}\leq$$\overset{N}{\sum_{j=0}}\alpha_{j}x_{{}_{ij}}+\left|F_{m}^{-1}\right|\overset{N}{\sum_{j=0}}c_{j}\left|x_{ij}\right|,$$y_{i}\geq$$\overset{N}{\sum_{j=0}}\alpha_{j}x_{{}_{ij}}-\left|F_{m}^{-1}\right|\overset{N}{\sum_{j=0}}c_{j}\left|x_{ij}\right|,\;$$x_{i0}=1,c_{j}\geq 0$where *F* is the membership function of a standardized fuzzy parameter [43].

Peters [5] modified Tanak’s model [1], introducing a new variable λ in the following way:

$$\max\bar{\lambda }=\frac{1}{M}\overset{M}{\sum_{i=1}}\lambda_{i}$$

with the constraints

$$\begin{array}{c} -d_{0}\leq \left(1-\bar{\lambda }\right)p_{0}-\sum_{i=0}^{M}\sum_{j=0}^{N}c_{j}\left|x_{ij}\right|,\\ -y_{i}\leq \left(1-\lambda_{i}\right)p_{i}-\sum_{j=0}^{N}\alpha_{j}x_{{}_{ij}}+\sum_{j=0}^{N}c_{j}\left|x_{ij}\right|,\\ y_{i}\leq \left(1-\lambda_{i}\right)p_{i}+\sum_{j=0}^{N}\alpha_{j}x_{{}_{ij}}+\sum_{j=0}^{N}c_{j}\left|x_{ij}\right|,\\ \;x_{i0}=1,c_{j}\geq 0,\left|F_{m}^{-1}\left(H\right)\right|=1, \end{array}$$

where λ($0\leq \lambda_{i}\leq 1$) represents the degree of membership that belongs to a set of good solutions.

The parameters $d_{0},\;p_{{}_{0}},and\;p_{{}_{i}}$ are selected to determine the width of the estimated interval. If a wide interval (a high $p_{0}$ and a small $p_{i}$) is deemed to minimize the spread, the requirement is regarded as lenient, while a narrow interval (a small $p_{0}$ and a high $p_{i}$) is taken as a strict condition. The value of $d_{{}_{0}}$, a desired value of the objective function, is taken as 0 [5].

Motivated by Peters [5], we introduced the PHFLRM for solving decision-making problems. The output variable $Y_{i}=\left\{y_{i}^{k}\left(p_{y_{i}^{k}}\right)|1<i<M,1<k<P\right\}$ and the input variables $X_{j}=\left\{x_{ij}^{k}\left(p_{x_{ij}^{k}}\right)|1<i<M,0<j<N,1<k<P\right\}$ are PHFEs. It is defined as:

$$Y_{i}={\tilde{\gamma }}_{0}⊙X_{0}⊕{\tilde{\gamma }}_{1}⊙X_{1}⊕{\tilde{\gamma }}_{2}⊙X_{2}⊕{\tilde{\gamma }}_{3}⊙X_{3}⊕\cdots ⊕{\tilde{\gamma }}_{N}⊙X_{N}$$

where the parameters ${\tilde{\gamma }}_{j}=\left(\alpha_{j}^{k},c_{j}^{k}\right),$ 0<j<N are symmetrical TFNs and *k* denotes the number of values assigned by the *P* DMs to the IPOP variables. The PHFLRM parameters are estimated using the following LPM.

$$\max\bar{\lambda^{k}}=\frac{1}{M}\overset{M}{\underset{i=1}{\sum \lambda_{i}^{k}}}$$

with the constraints

$$\begin{array}{c} -d_{0}\leq \left(1-\bar{\lambda^{k}}\right)p_{0}+\sum_{i=1}^{M}\sum_{j=0}^{N}c_{j}^{k}\left|x_{ij}^{k}\left(p_{x_{ij}^{k}}\right)\right|,\;\\ y_{i}^{k}\left(p_{y_{i}^{k}}\right)\leq \left(1-\lambda_{i}^{k}\right)p_{i}+\sum_{j=0}^{N}\alpha_{j}^{k}x_{ij}^{k}\left(p_{x_{ij}^{k}}\right)+\sum_{j=0}^{N}c_{j}^{k}\left|x_{ij}^{k}\left(p_{x_{ij}^{k}}\right)\right|,\\ -y_{i}^{k}\left(p_{y_{i}^{k}}\right)\leq \left(1-\lambda_{i}^{k}\right)p_{i}-\sum_{j=0}^{N}\alpha_{j}^{k}x_{ij}^{k}\left(p_{x_{ij}^{k}}\right)+\sum_{j=0}^{N}c_{j}^{k}\left|x_{ij}^{k}\left(p_{x_{ij}^{k}}\right)\right|,\\ \lambda_{i}^{k}\leq 1,\;\;x_{i0}^{k}\left(p_{x_{i0}^{k}}\right)=1,\;c_{j}^{k}\geq 0. \end{array}$$

## 4. Decision-Making Algorithms

In this section, we will describe the algorithms that are used to solve the PHFLRM and the TOPSIS method, respectively, in detail.

### 4.1. Algorithm for PHFLRM

**Figure 1:**
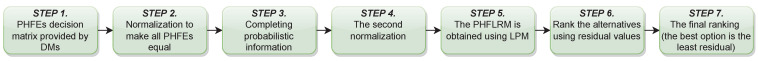
Flowchart of the proposed algorithm for PHFLRM.

Assume $A=\{A_{1},A_{2},\cdots ,A_{M}\}$ is a set of alternatives and $D=\{d^{l},1<l<P\}$ is a set of DMs that provide their evaluations in the form of PHFEs about alternatives $A_{i}$ under some input variables $X_{j}$(j=0,1,2,⋯,N) and output variable $Y_{i}$(i=1,2,⋯,M). Let $H_{1}={\left[X_{ij}\right]}_{M\times N}$ be an input variable decision matrix, $H_{2}={\left[Y_{i}\right]}_{M\times 1}$ be an output variable decision matrix, where $X_{ij}=\left\{x_{ij}^{k}\left(p_{x_{ij}^{k}}\right),\;k=1,2,\cdots ,\#\left(X_{ij}\right)\right\}$ and $Y_{i}=\{y_{i}^{k}\left(p_{y_{i}^{k}}\right),$$k=1,2,\cdots ,\#(Y_{i})\}$ are PHFEs. Figure 1 shows the flowchart of the proposed algorithm, and below are the detailed steps of this algorithm.

**Step** **1.** Let $H={\left[Z_{ij}\right]}_{M\times (N+1)}$ be a connected IPOP variable decision matrix provided by the DMs, where $Z_{ij}=\left\{z_{ij}^{k}\left(p_{z_{ij}^{k}}\right),\;k=1,2,\cdots ,\#\left(Z_{ij}\right)\right\}$ are PHFEs.
**Step** **2.** For two finite PHFEs $h_{1}$ and $h_{2}$, there are two opposite principles for normalization. The first one is α− normalization, in which we remove some elements of $h_{1}$ and $h_{2}$ which have more elements than the other ones. The second one is β− normalization, in which we add some elements to $h_{1}$ and $h_{2}\;$, which have fewer elements than the other one. In this study, we use the principle of β−normalization to make all PHFEs equal in the matrix *H*. Let $\overset{`}{H}={\left[{\overset{`}{Z}}_{ij}\right]}_{M\times (N+1)}$ be the normalized matrix, where ${\overset{`}{Z}}_{ij}=\left\{{\overset{`}{z}}_{ij}^{k}\left(p_{\bar{z}k}\right),\;k=1,2,\cdots ,S\right\}$ are PHFEs.
**Step** **3.** Using Definition 3, probabilistic information is completed for the PHFES in the decision matrix $\overset{`}{H}\;$. Let $\bar{H}={\left[{\bar{Z}}_{ij}\right]}_{M\times (N+1)}$ be a decision matrix after completing probabilistic information in the matrix $\overset{`}{H}$, where ${\bar{Z}}_{ij}=\left\{{\bar{z}}_{ij}^{k}\left(p_{\bar{z}k}\right),\;k=1,2,\cdots ,P\right\}$ are PHFEs.
**Step** **4.** Again, normalize the matrix $\bar{H}$ by using the following equation
  $${\hat{Z}}_{ij}=\frac{{\bar{z}}_{ij}^{k}p_{{\bar{z}}_{ij}^{k}}-\min\left({\bar{Z}}_{ij}\right)}{\max\left({\bar{Z}}_{ij}\right)-\min\left({\bar{Z}}_{ij}\right)}$$
  Let $\hat{H}={\left[{\hat{Z}}_{ij}\right]}_{M\times (N+1)}$ be a normalized decision matrix where ${\hat{Z}}_{ij}=\{{\hat{z}}_{ij}^{k}p_{{\hat{z}}_{ij}^{k}},$k=1,2,⋯,P} are PHFEs.
**Step** **5.** By using the normalized decision matrix $\hat{H}$, the PHFLRM is obtained. We further estimate the parameters of PHFLRM employing LPM.
**Step** **6.** Rank the alternatives using residual values obtained from the score values of $Y_{i}\left(i=1,2,\cdots ,M\right)$ and $Y_{i}^{*}\left(i=1,2,\cdots ,M\right)$ i.e.,$\;e_{i}=$$S_{c}\left(Y_{i}\right)-S_{c}\left(Y_{i}^{*}\right)$, where $Y_{i}^{*}$ are predicted values which are calculated by using Definitions 3 and 4.
**Step** **7.** Finally, the alternatives are ranked according to the values of $e_{i}\left(i=1,2,\cdots ,M\right)$. The alternative with the least residual is identified as the best choice.

The HFS, an extension of FST, has attracted the attention of many researchers in a short period, as hesitant situations are very common in real-world problems. Numerous extensions are introduced to address the uncertainty caused by hesitation; PHFS is one of them. The PHFS illustrates not only decision-makers’ hesitancy when they are undecided about something, but also the hesitant distribution of information. In the PHFLRM (3) IPOP variables are observed as PHFEs instead HFEs, which is a basic form of PHFS.

### 4.2. The TOPSIS Algorithm

A MCDM methodology, TOPSIS, was developed by Hwang and Yoon [30], which provides the shortest distance from the positive ideal solution (PIS) and the longest from the negative ideal solution (NIS) for all possible alternatives. The mathematical formulation of the TOPSIS method when the criteria values are PHFEs is as follows:

**Step** **1.** Take the decision matrices $H,\;\overset{`}{H}$ and $\bar{H}$ same as mentioned in Step 1, 2, and 3 of Section 4.1.
**Step** **2.** Normalize the decision matrix $\bar{H}$ with the help of the following formula.
  $${\hat{Z}}_{ij}=\left\{\frac{z_{11}^{1}p_{z_{11}^{1}}^{1}}{\underset{i=1}{\sum^{M}}{\left(z_{i1}^{1}p_{z_{11}^{1}}^{1}\right)}^{2}},\frac{z_{11}^{2}p_{z_{12}^{2}}^{2}}{\underset{i=1}{\sum^{M}}{\left(z_{i2}^{2}p_{z_{i2}^{2}}^{2}\right)}^{2}},\cdots ,\frac{z_{1(N+1)}^{P}p_{z_{1(N+1)}^{P}}^{P}}{\underset{i=1}{\sum^{M}}{\left(z_{i(N+1)}^{P}p_{z_{i(N+1)}^{P}}^{P}\right)}^{2}}\right\}$$
  Let $\hat{H}={\left[{\hat{Z}}_{ij}\right]}_{M\times (N+1)}$ be the normalized decision matrix, where ${\hat{Z}}_{ij}$ are PHFEs.
**Step** **3.** The weighted normalized decision matrix is calculated by multiplaying the normalized decision matrix with its associated weights, i.e., $V_{ij}={\hat{Z}}_{ij}\times W_{j}$, where $V_{ij}$ is a PHFE.
**Step** **4.** Determine the positive ideal solution $A^{+}$ and negative ideal solution $A^{-}$ as
  $$\begin{array}{ccc} A^{+} & = & \{\left(\max_{i}V_{ij}|j\in J_{b}\right),(\min_{i}V_{ij}|j\in J_{c}\left|i=1,2,\cdots ,N)\right\}\\ & = & \left\{A_{1}^{+},A_{2}^{+},\cdots ,A_{J}^{+},\cdots ,A_{\left(N+1\right)}\right\}\\ A^{-} & = & \{\left(\min_{i}V_{ij}|j\in J_{b}\right),(\max_{i}V_{ij}|j\in J_{c}\left|i=1,2,\cdots ,N)\right\}\\ & = & \left\{A_{1}^{-},A_{2}^{-},\cdots ,A_{J}^{-},\cdots ,A_{{\left(N+1\right)}^{-}}^{-}\right\} \end{array}$$
  where $J_{b}$ and $J_{c}$ represent the set of benefit and cost criteria, respectively.
**Step** **5.** Calculate the Euclidean distances of D${}_{i}^{+}$ and D${}_{i}^{-}\;$ of each alternative A${}_{i}$ from the positive ideal solution A${}^{+}$ and negative ideal solution A${}^{-}$, respectively, by using Definition 6.
**Step** **6.** Calculate the relative closeness $P_{i}\;$of each alternative to the ideal solution as
  $$P_{i}=\frac{D_{i}^{-}}{D_{i}^{-}+D_{i}^{+}},i=1,2,\cdots ,M.\;$$
**Step** **7.** The alternatives $A_{i}\left(i=1,2,\cdots ,M\right)\;$ are ranked according to relative closeness values $P_{i}$ in the descending order.

## 5. Application Example

Wheat is the most important rabi crop in Pakistan, and it is also the country’s staple diet. Wheat production is one of the most pressing concerns confronting the agricultural industry today, and it is expected to continue to grow. Various factors such as farm size, seed quality, fertilizer price, irrigation area, and rain amount contribute to the yield of wheat. In this example, a simultaneous analysis including multiple variables is performed for efficient decision making. We consider rain amount $(X_{1}),\;$farm size$\;(X_{2})$, and irrigation area $\left(X_{3}\right)$ in order to determine their effect on wheat yield (Y). Twelve districts $A_{i}$(i=1,2,⋯,12) of Punjab (Pakistan) are selected in the form of alternatives. These alternatives are evaluated using Yi(i=1,2,⋯,12) and Xj+1(j=0,1,2) as input and output variables, respectively. The IPOP variables have been evaluated by three agriculture department experts. The steps necessary to resolve this problem are listed below.

**Step** **1.** Table 1 shows the connected IPOP variable decision matrix provided by the DMs employing PHFEs.To make all PHFES equal using beta−normalization and to make the sum of all probabilities equal to one for all PHFES in the decision matrix *H* and $\overset{`}{H}$, respectively, and to obtain matrix $\bar{H}$, as shown in Table 2.
**Step** **2** **&** **3.** We obtain the matrix $\bar{H}$, which can be shown in Table 2, by making all PHFES equal using beta−normalization and making the sum of all probabilities equal to one for all PHFES in the decision matrix *H* and $\overset{`}{H}$, respectively.
**Step** **4** **&** **5.** We will estimate the parameters from the normalized decision matrix $\hat{H}$ using LP after normalizing the matrix $\bar{H}$, as follows:

For k=1

$$\mathrm{Max}{\bar{\lambda }}^{1}=\frac{\underset{i=1}{\overset{M}{\sum \lambda^{1}}}}{M}$$

Subject to the constraints

$$\left(\lambda_{1}^{1}+\lambda_{2}^{1}+\ldots +\lambda_{12}^{1}\right)+\frac{12}{1000}(12c_{0}^{1}+5.0417c_{1}^{1}+0.3597c_{2}^{1}+0.0534c_{3}^{1}\leq 12$$

and

$$\begin{array}{c} \lambda_{1}^{1}-\left(\alpha_{0}^{1}+0.4398\alpha_{1}^{1}+0.0121\alpha_{2}^{1}+0.0073\alpha_{3}^{1}\right)-\left(c_{0}^{1}+0.4398c_{1}^{1}+0.0121c_{2}^{1}+0.0073c_{3}^{1}\right)\leq 0.2416\\ \lambda_{2}^{1}-\left(\alpha_{0}^{1}+0.3254\alpha_{1}^{1}+0.0277\alpha_{2}^{1}+0.0040\alpha_{3}^{1}\right)-\left(c_{0}^{1}+0.3254c_{1}^{1}+0.0277c_{2}^{1}+0.0040c_{3}^{1}\right)\leq 0.1681\\ \lambda_{3}^{1}-\left(\alpha_{0}^{1}+0.5052\alpha_{1}^{1}+0.0173\alpha_{2}^{1}+0.0028\alpha_{3}^{1}\right)-\left(c_{0}^{1}+0.5052c_{1}^{1}+0.0173c_{2}^{1}+0.0028c_{3}^{1}\right)\leq 0.3053\\ \lambda_{4}^{1}-\left(\alpha_{0}^{1}+0.4251\alpha_{1}^{1}+0.0290\alpha_{2}^{1}+0.0026\alpha_{3}^{1}\right)-\left(c_{0}^{1}+0.4251c_{1}^{1}+0.0290c_{2}^{1}+0.0026c_{3}^{1}\right)\leq 0.1061\\ \lambda_{5}^{1}-\left(\alpha_{0}^{1}+0.4914\alpha_{1}^{1}+0.0248\alpha_{2}^{1}+0.0079\alpha_{3}^{1}\right)-\left(c_{0}^{1}+0.4914c_{1}^{1}+0.0248c_{2}^{1}+0.0079c_{3}^{1}\right)\leq 0.0915\\ \lambda_{6}^{1}-\left(\alpha_{0}^{1}+0.4447\alpha_{1}^{1}+0.0264\alpha_{2}^{1}+0.0052\alpha_{3}^{1}\right)-\left(c_{0}^{1}+0.4447c_{1}^{1}+0.0264c_{2}^{1}+0.0052c_{3}^{1}\right)\leq 0.0467\\ \lambda_{7}^{1}-\left(\alpha_{0}^{1}+0.4643\alpha_{1}^{1}+0.0305\alpha_{2}^{1}+0.0055\alpha_{3}^{1}\right)-\left(c_{0}^{1}+0.4643c_{1}^{1}+0.0305c_{2}^{1}+0.0055c_{3}^{1}\right)\leq 0.1354\\ \lambda_{8}^{1}-\left(\alpha_{0}^{1}+0.3590\alpha_{1}^{1}+0.0379\alpha_{2}^{1}+0.0089\alpha_{3}^{1}\right)-\left(c_{0}^{1}+0.3590c_{1}^{1}+0.0379c_{2}^{1}+0.0089c_{3}^{1}\right)\leq 0.0280\\ \lambda_{9}^{1}-\left(\alpha_{0}^{1}+0.4002\alpha_{1}^{1}+0.0460\alpha_{2}^{1}+0.0071\alpha_{3}^{1}\right)-\left(c_{0}^{1}+0.4002c_{1}^{1}+0.0460c_{2}^{1}+0.0071c_{3}^{1}\right)\leq 0.2544\\ \lambda_{10}^{1}-\left(\alpha_{0}^{1}+0.4290\alpha_{1}^{1}+0.0310\alpha_{2}^{1}+0.0011\alpha_{3}^{1}\right)-\left(c_{0}^{1}+0.4290c_{1}^{1}+0.0011c_{2}^{1}+0.0243c_{3}^{1}\right)\leq 0.0244\\ \lambda_{11}^{1}-\left(\alpha_{0}^{1}+0.4067\alpha_{1}^{1}+0.0513\alpha_{2}^{1}+0.0010\alpha_{3}^{1}\right)-\left(c_{0}^{1}+0.4067c_{1}^{1}+0.0513c_{2}^{1}+0.0010c_{3}^{1}\right)\leq 0.1288\\ \lambda_{12}^{1}-\left(\alpha_{0}^{1}+0.3509\alpha_{1}^{1}+0.0257\alpha_{2}^{1}+0.0000\alpha_{3}^{1}\right)-\left(c_{0}^{1}+0.3509c_{1}^{1}+0.0257c_{2}^{1}+0.0000c_{3}^{1}\right)\leq 0.3132 \end{array}$$

and

$$\begin{array}{c} \lambda_{1}^{1}+\left(\alpha_{0}^{1}+0.4398\alpha_{1}^{1}+0.0121\alpha_{2}^{1}+0.0073\alpha_{3}^{1}\right)-\left(c_{0}^{1}+0.4398c_{1}^{1}+0.0121c_{2}^{1}+0.0073c_{3}^{1}\right)\leq 1.7584\\ \lambda_{2}^{1}+\left(\alpha_{0}^{1}+0.3254\alpha_{1}^{1}+0.0277\alpha_{2}^{1}+0.0040\alpha_{3}^{1}\right)-\left(c_{0}^{1}+0.3254c_{1}^{1}+0.0277c_{2}^{1}+0.0040c_{3}^{1}\right)\leq 1.8319\\ \lambda_{3}^{1}+\left(\alpha_{0}^{1}+0.5052\alpha_{1}^{1}+0.0173\alpha_{2}^{1}+0.0028\alpha_{3}^{1}\right)-\left(c_{0}^{1}+0.5052c_{1}^{1}+0.0173c_{2}^{1}+0.0028c_{3}^{1}\right)\leq 1.6947\\ \lambda_{4}^{1}+\left(\alpha_{0}^{1}+0.4251\alpha_{1}^{1}+0.0290\alpha_{2}^{1}+0.0026\alpha_{3}^{1}\right)-\left(c_{0}^{1}+0.4251c_{1}^{1}+0.0290c_{2}^{1}+0.0026c_{3}^{1}\right)\leq 1.8939\\ \lambda_{5}^{1}+\left(\alpha_{0}^{1}+0.4914\alpha_{1}^{1}+0.0248\alpha_{2}^{1}+0.0079\alpha_{3}^{1}\right)-\left(c_{0}^{1}+0.4914c_{1}^{1}+0.0248c_{2}^{1}+0.0079c_{3}^{1}\right)\leq 1.9085\\ \lambda_{6}^{1}+\left(\alpha_{0}^{1}+0.4447\alpha_{1}^{1}+0.0264\alpha_{2}^{1}+0.0052\alpha_{3}^{1}\right)-\left(c_{0}^{1}+0.4447c_{1}^{1}+0.0264c_{2}^{1}+0.0052c_{3}^{1}\right)\leq 1.9533\\ \lambda_{7}^{1}+\left(\alpha_{0}^{1}+0.4643\alpha_{1}^{1}+0.0305\alpha_{2}^{1}+0.0055\alpha_{3}^{1}\right)-\left(c_{0}^{1}+0.4643c_{1}^{1}+0.0305c_{2}^{1}+0.0055c_{3}^{1}\right)\leq 1.8646\\ \lambda_{8}^{1}+\left(\alpha_{0}^{1}+0.3590\alpha_{1}^{1}+0.0379\alpha_{2}^{1}+0.0089\alpha_{3}^{1}\right)-\left(c_{0}^{1}+0.3590c_{1}^{1}+0.0379c_{2}^{1}+0.0089c_{3}^{1}\right)\leq 1.9720\\ \lambda_{9}^{1}+\left(\alpha_{0}^{1}+0.4002\alpha_{1}^{1}+0.0460\alpha_{2}^{1}+0.0071\alpha_{3}^{1}\right)-\left(c_{0}^{1}+0.4002c_{1}^{1}+0.0460c_{2}^{1}+0.0071c_{3}^{1}\right)\leq 1.7456\\ \lambda_{10}^{1}+\left(\alpha_{0}^{1}+0.4290\alpha_{1}^{1}+0.0310\alpha_{2}^{1}+0.0011\alpha_{3}^{1}\right)-\left(c_{0}^{1}+0.4290c_{1}^{1}+0.0011c_{2}^{1}+0.0243c_{3}^{1}\right)\leq 1.9757\\ \lambda_{11}^{1}+\left(\alpha_{0}^{1}+0.4067\alpha_{1}^{1}+0.0513\alpha_{2}^{1}+0.0010\alpha_{3}^{1}\right)-\left(c_{0}^{1}+0.4067c_{1}^{1}+0.0513c_{2}^{1}+0.0010c_{3}^{1}\right)\leq 1.8712\\ \lambda_{12}^{1}+\left(\alpha_{0}^{1}+0.3509\alpha_{1}^{1}+0.0257\alpha_{2}^{1}+0.0000\alpha_{3}^{1}\right)-\left(c_{0}^{1}+0.3509c_{1}^{1}+0.0257c_{2}^{1}+0.0000c_{3}^{1}\right)\leq 1.6868 \end{array}$$

For k=2

$$\mathrm{Max}{\bar{\lambda }}^{2}=\frac{\underset{i=1}{\overset{M}{\sum \lambda^{2}}}}{M}$$

Subject to the constraints

$$\left(\lambda_{1}^{2}+\lambda_{2}^{2}+\lambda_{3}^{2}+\ldots +\lambda_{12}^{2}\right)+\frac{12}{1000}(12c_{0}^{2}+5.0660c_{1}^{2}+0.3584c_{2}^{2}+0.0308c_{3}^{2}\leq 12$$

and

$$\begin{array}{c} \lambda_{1}^{2}-\left(\alpha_{0}^{2}+0.3758\alpha_{1}^{2}+0.0197\alpha_{2}^{2}+0.0012\alpha_{3}^{2}\right)-\left(c_{0}^{2}+0.3758c_{1}^{2}+0.0197c_{2}^{2}+0.0012c_{3}^{2}\right)\leq 0.3483\\ \lambda_{2}^{2}-\left(\alpha_{0}^{2}+0.3843\alpha_{1}^{2}+0.0228\alpha_{2}^{2}+0.0012\alpha_{3}^{2}\right)-\left(c_{0}^{2}+0.3843c_{1}^{2}+0.0228c_{2}^{2}+0.0012c_{3}^{2}\right)\leq 0.1615\\ \lambda_{3}^{2}-\left(\alpha_{0}^{2}+0.5068\alpha_{1}^{2}+0.0261\alpha_{2}^{2}+0.0030\alpha_{3}^{2}\right)-\left(c_{0}^{2}+0.5052c_{1}^{2}+0.0261c_{2}^{2}+0.0030c_{3}^{2}\right)\leq 0.3020\\ \lambda_{4}^{2}-\left(\alpha_{0}^{2}+0.3632\alpha_{1}^{2}+0.0187\alpha_{2}^{2}+0.0018\alpha_{3}^{2}\right)-\left(c_{0}^{2}+0.3632c_{1}^{2}+0.0018c_{2}^{2}+0.0026c_{3}^{2}\right)\leq 0.2275\\ \lambda_{5}^{2}-\left(\alpha_{0}^{2}+0.4201\alpha_{1}^{2}+0.0264\alpha_{2}^{2}+0.0016\alpha_{3}^{2}\right)-\left(c_{0}^{2}+0.0264c_{1}^{2}+0.0248c_{2}^{2}+0.0016c_{3}^{2}\right)\leq 0.3174\\ \lambda_{6}^{2}-\left(\alpha_{0}^{2}+0.4463\alpha_{1}^{2}+0.0281\alpha_{2}^{2}+0.0052\alpha_{3}^{2}\right)-\left(c_{0}^{2}+0.4463c_{1}^{2}+0.0281c_{2}^{2}+0.0052c_{3}^{2}\right)\leq 0.2810\\ \lambda_{7}^{2}-\left(\alpha_{0}^{2}+0.3969\alpha_{1}^{2}+0.0236\alpha_{2}^{2}+0.0056\alpha_{3}^{2}\right)-\left(c_{0}^{2}+0.3969c_{1}^{2}+0.0236c_{2}^{2}+0.0056c_{3}^{2}\right)\leq 0.1272\\ \lambda_{8}^{2}-\left(\alpha_{0}^{2}+0.4235\alpha_{1}^{2}+0.0379\alpha_{2}^{2}+0.0024\alpha_{3}^{2}\right)-\left(c_{0}^{2}+0.4235c_{1}^{2}+0.0379c_{2}^{2}+0.0024c_{3}^{2}\right)\leq 0.0000\\ \lambda_{9}^{2}-\left(\alpha_{0}^{2}+0.3509\alpha_{1}^{2}+0.0383\alpha_{2}^{2}+0.0012\alpha_{3}^{2}\right)-\left(c_{0}^{2}+0.3509c_{1}^{2}+0.0383c_{2}^{2}+0.0012c_{3}^{2}\right)\leq 0.1223\\ \lambda_{10}^{2}-\left(\alpha_{0}^{2}+0.5052\alpha_{1}^{2}+0.0408\alpha_{2}^{2}+0.0043\alpha_{3}^{2}\right)-\left(c_{0}^{2}+0.5052c_{1}^{2}+0.0408c_{2}^{2}+0.0043c_{3}^{2}\right)\leq 0.0149\\ \lambda_{11}^{2}-\left(\alpha_{0}^{2}+0.4790\alpha_{1}^{2}+0.0414\alpha_{2}^{2}+0.0011\alpha_{3}^{2}\right)-\left(c_{0}^{2}+0.4790c_{1}^{2}+0.0414c_{2}^{2}+0.0011c_{3}^{2}\right)\leq 0.2474\\ \lambda_{12}^{2}-\left(\alpha_{0}^{2}+0.4140\alpha_{1}^{2}+0.0048\alpha_{2}^{2}+0.0000\alpha_{3}^{2}\right)-\left(c_{0}^{2}+0.4140c_{1}^{2}+0.0346c_{2}^{2}+0.0048c_{3}^{2}\right)\leq 0.1893 \end{array}$$

and

$$\begin{array}{c} \lambda_{1}^{2}+\left(\alpha_{0}^{2}+0.3758\alpha_{1}^{2}+0.0197\alpha_{2}^{2}+0.0012\alpha_{3}^{2}\right)-\left(c_{0}^{2}+0.3758c_{1}^{2}+0.0197c_{2}^{2}+0.0012c_{3}^{2}\right)\leq 1.6517\\ \lambda_{2}^{2}+\left(\alpha_{0}^{2}+0.3843\alpha_{1}^{2}+0.0228\alpha_{2}^{2}+0.0012\alpha_{3}^{2}\right)-\left(c_{0}^{2}+0.3843c_{1}^{2}+0.0228c_{2}^{2}+0.0012c_{3}^{2}\right)\leq 1.8385\\ \lambda_{3}^{2}+\left(\alpha_{0}^{2}+0.5068\alpha_{1}^{2}+0.0261\alpha_{2}^{2}+0.0030\alpha_{3}^{2}\right)-\left(c_{0}^{2}+0.5052c_{1}^{2}+0.0261c_{2}^{2}+0.0030c_{3}^{2}\right)\leq 1.6980\\ \lambda_{4}^{2}+\left(\alpha_{0}^{2}+0.3632\alpha_{1}^{2}+0.0187\alpha_{2}^{2}+0.0018\alpha_{3}^{2}\right)-\left(c_{0}^{2}+0.3632c_{1}^{2}+0.0018c_{2}^{2}+0.0026c_{3}^{2}\right)\leq 1.7725\\ \lambda_{5}^{2}+\left(\alpha_{0}^{2}+0.4201\alpha_{1}^{2}+0.0264\alpha_{2}^{2}+0.0016\alpha_{3}^{2}\right)-\left(c_{0}^{2}+0.0264c_{1}^{2}+0.0248c_{2}^{2}+0.0016c_{3}^{2}\right)\leq 1.6826\\ \lambda_{6}^{2}+\left(\alpha_{0}^{2}+0.4463\alpha_{1}^{2}+0.0281\alpha_{2}^{2}+0.0052\alpha_{3}^{2}\right)-\left(c_{0}^{2}+0.4463c_{1}^{2}+0.0281c_{2}^{2}+0.0052c_{3}^{2}\right)\leq 1.7190\\ \lambda_{7}^{2}+\left(\alpha_{0}^{2}+0.3969\alpha_{1}^{2}+0.0236\alpha_{2}^{2}+0.0056\alpha_{3}^{2}\right)-\left(c_{0}^{2}+0.3969c_{1}^{2}+0.0236c_{2}^{2}+0.0056c_{3}^{2}\right)\leq 1.8728\\ \lambda_{8}^{2}+\left(\alpha_{0}^{2}+0.4235\alpha_{1}^{2}+0.0379\alpha_{2}^{2}+0.0024\alpha_{3}^{2}\right)-\left(c_{0}^{2}+0.4235c_{1}^{2}+0.0379c_{2}^{2}+0.0024c_{3}^{2}\right)\leq 2.0000\\ \lambda_{9}^{2}+\left(\alpha_{0}^{2}+0.3509\alpha_{1}^{2}+0.0383\alpha_{2}^{2}+0.0012\alpha_{3}^{2}\right)-\left(c_{0}^{2}+0.3509c_{1}^{2}+0.0383c_{2}^{2}+0.0012c_{3}^{2}\right)\leq 1.8777\\ \lambda_{10}^{2}+\left(\alpha_{0}^{2}+0.5052\alpha_{1}^{2}+0.0408\alpha_{2}^{2}+0.0043\alpha_{3}^{2}\right)-\left(c_{0}^{2}+0.5052c_{1}^{2}+0.0408c_{2}^{2}+0.0043c_{3}^{2}\right)\leq 1.9851\\ \lambda_{11}^{2}+\left(\alpha_{0}^{2}+0.4790\alpha_{1}^{2}+0.0414\alpha_{2}^{2}+0.0011\alpha_{3}^{2}\right)-\left(c_{0}^{2}+0.4790c_{1}^{2}+0.0414c_{2}^{2}+0.0011c_{3}^{2}\right)\leq 1.7526\\ \lambda_{12}^{2}+\left(\alpha_{0}^{2}+0.4140\alpha_{1}^{2}+0.0048\alpha_{2}^{2}+0.0000\alpha_{3}^{2}\right)-\left(c_{0}^{2}+0.4140c_{1}^{2}+0.0346c_{2}^{2}+0.0048c_{3}^{2}\right)\leq 1.8107 \end{array}$$

For k=3

$$\mathrm{Max}{\bar{\lambda }}^{3}=\frac{\underset{i=1}{\overset{M}{\sum \lambda^{3}}}}{M}$$

Subject to the constraints

$$\left(\lambda_{1}^{3}+\lambda_{2}^{3}+\lambda_{3}^{3}+\ldots +\lambda_{12}^{3}\right)+\frac{12}{1000}(12c_{0}^{3}+5.0111c_{1}^{3}+0.3423c_{2}^{3}+0.0424c_{3}^{3}\leq 12$$

and

$$\begin{array}{c} \lambda_{1}^{3}-\left(\alpha_{0}^{3}+0.4431\alpha_{1}^{3}+0.0277\alpha_{2}^{3}+0.0012\alpha_{3}^{3}\right)-\left(c_{0}^{3}+0.4431c_{1}^{3}+0.0277c_{2}^{3}+0.0012c_{3}^{3}\right)\leq 0.2301\\ \lambda_{2}^{3}-\left(\alpha_{0}^{3}+0.3859\alpha_{1}^{3}+0.0173\alpha_{2}^{3}+0.0045\alpha_{3}^{3}\right)-\left(c_{0}^{3}+0.3859c_{1}^{3}+0.0173c_{2}^{3}+0.0045c_{3}^{3}\right)\leq 0.2754\\ \lambda_{3}^{3}-\left(\alpha_{0}^{3}+0.4333\alpha_{1}^{3}+0.0202\alpha_{2}^{3}+0.0001\alpha_{3}^{3}\right)-\left(c_{0}^{3}+0.4333c_{1}^{3}+0.0202c_{2}^{3}+0.0001c_{3}^{3}\right)\leq 0.4029\\ \lambda_{4}^{3}-\left(\alpha_{0}^{3}+0.4284\alpha_{1}^{3}+0.0243\alpha_{2}^{3}+0.0052\alpha_{3}^{3}\right)-\left(c_{0}^{3}+0.4284c_{1}^{3}+0.0243c_{2}^{3}+0.0052c_{3}^{3}\right)\leq 0.2216\\ \lambda_{5}^{3}-\left(\alpha_{0}^{3}+0.4216\alpha_{1}^{3}+0.0201\alpha_{2}^{3}+0.0016\alpha_{3}^{3}\right)-\left(c_{0}^{3}+0.4216c_{1}^{3}+0.0201c_{2}^{3}+0.0016c_{3}^{3}\right)\leq 0.3090\\ \lambda_{6}^{3}-\left(\alpha_{0}^{3}+0.3815\alpha_{1}^{3}+0.0229\alpha_{2}^{3}+0.0065\alpha_{3}^{3}\right)-\left(c_{0}^{3}+0.3815c_{1}^{3}+0.0229c_{2}^{3}+0.0065c_{3}^{3}\right)\leq 0.2754\\ \lambda_{7}^{3}-\left(\alpha_{0}^{3}+0.4676\alpha_{1}^{3}+0.0265\alpha_{2}^{3}+0.0023\alpha_{3}^{3}\right)-\left(c_{0}^{3}+0.4676c_{1}^{3}+0.0265c_{2}^{3}+0.0023c_{3}^{3}\right)\leq 0.2460\\ \lambda_{8}^{3}-\left(\alpha_{0}^{3}+0.4251\alpha_{1}^{3}+0.0313\alpha_{2}^{3}+0.0025\alpha_{3}^{3}\right)-\left(c_{0}^{3}+0.4251c_{1}^{3}+0.0313c_{2}^{3}+0.0025c_{3}^{3}\right)\leq 0.5023\\ \lambda_{9}^{3}-\left(\alpha_{0}^{3}+0.3582\alpha_{1}^{3}+0.0477\alpha_{2}^{3}+0.0014\alpha_{3}^{3}\right)-\left(c_{0}^{3}+0.3582c_{1}^{3}+0.0477c_{2}^{3}+0.0014c_{3}^{3}\right)\leq 0.1190\\ \lambda_{10}^{3}-\left(\alpha_{0}^{3}+0.4319\alpha_{1}^{3}+0.0339\alpha_{2}^{3}+0.0046\alpha_{3}^{3}\right)-\left(c_{0}^{3}+0.4319c_{1}^{3}+0.0339c_{2}^{3}+0.0046c_{3}^{3}\right)\leq 0.5135\\ \lambda_{11}^{3}-\left(\alpha_{0}^{3}+0.4807\alpha_{1}^{3}+0.0342\alpha_{2}^{3}+0.0076\alpha_{3}^{3}\right)-\left(c_{0}^{3}+0.4807c_{1}^{3}+0.0342c_{2}^{3}+0.0076c_{3}^{3}\right)\leq 0.1141\\ \lambda_{12}^{3}-\left(\alpha_{0}^{3}+0.3538\alpha_{1}^{3}+0.0362\alpha_{2}^{3}+0.0049\alpha_{3}^{3}\right)-\left(c_{0}^{3}+0.3538c_{1}^{3}+0.0362c_{2}^{3}+0.0049c_{3}^{3}\right)\leq 0.1877 \end{array}$$

and

$$\begin{array}{c} \lambda_{1}^{3}+\left(\alpha_{0}^{3}+0.4431\alpha_{1}^{3}+0.0277\alpha_{2}^{3}+0.0012\alpha_{3}^{3}\right)-\left(c_{0}^{3}+0.4431c_{1}^{3}+0.0277c_{2}^{3}+0.0012c_{3}^{3}\right)\leq 1.7699\\ \lambda_{2}^{3}+\left(\alpha_{0}^{3}+0.3859\alpha_{1}^{3}+0.0173\alpha_{2}^{3}+0.0045\alpha_{3}^{3}\right)-\left(c_{0}^{3}+0.3859c_{1}^{3}+0.0173c_{2}^{3}+0.0045c_{3}^{3}\right)\leq 1.7246\\ \lambda_{3}^{3}+\left(\alpha_{0}^{3}+0.4333\alpha_{1}^{3}+0.0202\alpha_{2}^{3}+0.0001\alpha_{3}^{3}\right)-\left(c_{0}^{3}+0.4333c_{1}^{3}+0.0202c_{2}^{3}+0.0001c_{3}^{3}\right)\leq 1.5971\\ \lambda_{4}^{3}+\left(\alpha_{0}^{3}+0.4284\alpha_{1}^{3}+0.0243\alpha_{2}^{3}+0.0052\alpha_{3}^{3}\right)-\left(c_{0}^{3}+0.4284c_{1}^{3}+0.0243c_{2}^{3}+0.0052c_{3}^{3}\right)\leq 1.7784\\ \lambda_{5}^{3}+\left(\alpha_{0}^{3}+0.4216\alpha_{1}^{3}+0.0201\alpha_{2}^{3}+0.0016\alpha_{3}^{3}\right)-\left(c_{0}^{3}+0.4216c_{1}^{3}+0.0201c_{2}^{3}+0.0016c_{3}^{3}\right)\leq 1.6910\\ \lambda_{6}^{3}+\left(\alpha_{0}^{3}+0.3815\alpha_{1}^{3}+0.0229\alpha_{2}^{3}+0.0065\alpha_{3}^{3}\right)-\left(c_{0}^{3}+0.3815c_{1}^{3}+0.0229c_{2}^{3}+0.0065c_{3}^{3}\right)\leq 1.7246\\ \lambda_{7}^{3}+\left(\alpha_{0}^{3}+0.4676\alpha_{1}^{3}+0.0265\alpha_{2}^{3}+0.0023\alpha_{3}^{3}\right)-\left(c_{0}^{3}+0.4676c_{1}^{3}+0.0265c_{2}^{3}+0.0023c_{3}^{3}\right)\leq 1.7540\\ \lambda_{8}^{3}+\left(\alpha_{0}^{3}+0.4251\alpha_{1}^{3}+0.0313\alpha_{2}^{3}+0.0025\alpha_{3}^{3}\right)-\left(c_{0}^{3}+0.4251c_{1}^{3}+0.0313c_{2}^{3}+0.0025c_{3}^{3}\right)\leq 1.4977\\ \lambda_{9}^{3}+\left(\alpha_{0}^{3}+0.3582\alpha_{1}^{3}+0.0477\alpha_{2}^{3}+0.0014\alpha_{3}^{3}\right)-\left(c_{0}^{3}+0.3582c_{1}^{3}+0.0477c_{2}^{3}+0.0014c_{3}^{3}\right)\leq 1.8810\\ \lambda_{10}^{3}+\left(\alpha_{0}^{3}+0.4319\alpha_{1}^{3}+0.0339\alpha_{2}^{3}+0.0046\alpha_{3}^{3}\right)-\left(c_{0}^{3}+0.4319c_{1}^{3}+0.0339c_{2}^{3}+0.0046c_{3}^{3}\right)\leq 1.4865\\ \lambda_{11}^{3}+\left(\alpha_{0}^{3}+0.4807\alpha_{1}^{3}+0.0342\alpha_{2}^{3}+0.0076\alpha_{3}^{3}\right)-\left(c_{0}^{3}+0.4807c_{1}^{3}+0.0342c_{2}^{3}+0.0076c_{3}^{3}\right)\leq 1.8859\\ \lambda_{12}^{3}+\left(\alpha_{0}^{3}+0.3538\alpha_{1}^{3}+0.0362\alpha_{2}^{3}+0.0049\alpha_{3}^{3}\right)-\left(c_{0}^{3}+0.3538c_{1}^{3}+0.0362c_{2}^{3}+0.0049c_{3}^{3}\right)\leq 1.8123 \end{array}$$

After solving the linear programming model, as mentioned above, we get the values of $\lambda_{i}^{k}\left(i=1,2,\cdots ,12\right)$,$\;\alpha_{j}^{k}\left(j=1,2,3,4\right)$ and $c_{j}^{k}\left(j=1,2,3,4\right)$, which are shown in Table 3:

We can see in Table 3 that the estimated values $\lambda_{i}^{k}$ obtained by solving a LP model are either equal to 1 or very close to 1. The resultant estimated PHFLRM employing equations from Section 3, is obtained as follows:

$$\begin{array}{ccc} Y^{*} & = & \left(0.6467,0.01252\right)⊕\left(-0.1575,0.06378\right)⊙X_{1}⊕\left(5.3685,2.6160\right)⊙X_{2}\\ & & ⊕\left(8.8450,0.4811\right)⊙X_{3}. \end{array}$$

**Step** **6** **&** **7.** By using PHFLRM, we will find the estimated PHFEs $(Y^{*})\;$ of all possible alternatives. To save time, we will just compute the estimated PHFE $Y_{1}^{*}$ against the alternative $A_{1}$ using the Definition 3 and 4, as follows:

$$\begin{array}{cc} Y_{1}^{*} & =\{0.7194(0.03889),0.7137(0.03333),0.7197(0.03888),0.7308(0.04667),\\ \; & \;0.7253(0.04667),0.7311(0.04667),0.7424(0.05444),0.7371(0.04667),\\ \; & \;0.7559(0.05444),0.7038(0.02917),0.6977(0.02500),0.7041(0.02917),\\ \; & \;0.7158(0.03500),0.7100(0.03000),0.7162(0.03500),0.7281(0.04083),\\ \; & \;0.7225(0.03500),0.7284(0.04083),0.7038(0.02917),0.6977(0.02500),\\ \; & \;0.7041(0.02917),0.7158(0.03500),0.7100(0.03000),0.7162(0.03500),\\ \; & \;0.7281(0.04083),0.7281(0.03500),0.7284(0.04083)\} \end{array}$$

By using Definition 5, the score value ($Sc(Y_{1}^{*})$) of the estimated PHFE ($Y_{1}^{*}$) is computed, i.e., 0.7223. In the same way, we can find all score values $(Sc\left(Y_{i}^{*}\right)$ of the estimated PHFEs, $Y_{i}^{*}\left(i=2,\cdots ,12\right)$, in Table 4. Further, residual values $e_{i}$ against each alternative $Y_{i}$ are calculated as $e_{i}=Sc\left(Y_{i}\right)-Sc\left(Y_{i}^{*}\right),\;i=1,2,\cdots ,12,$ and finally, all alternatives are ranked using these residual values $e_{i}$, in Table 4. We have the smallest residual $e_{3}=30.04827$ against the alternative $A_{{}_{3}}$, so it is considered as the best choice. Additionally, the alternative $A_{{}_{11}}$ has the largest residual $e_{11}=47.9838$, and is considered the worst alternative.

### A Comparative Study of the PHFLRM and the TOPSIS

The TOPSIS method, which is a MCDM tool, has been used to verify the results and efficiency of our proposed approach. For the same problem, the results of the proposed method are compared with the results of the TOPSIS method. We have taken rain amount $(X_{1}),\;$ farm size $\left(X_{2}\right)$, irrigated area $\;(X_{3})$, and wheat yield (Y) as the benefit criteria. Following steps 1,2,and3 of the TOPSIS algorithm (Section 4.1), we have the PIS $\left(A^{+}\right)$ and NIS $\left(A^{-}\right)$, as follows:

$$\begin{array}{c} A^{+}=\{\left\{0.3422,0.3420,0.3292\right\},\left\{0.4079,0.3542.0.4019\right\},\\ \left\{0.3437,0.3312,0.3420\},\{0.3297,0.3540,0.3500\}\right\}\\ A^{-}=\{\left\{0.2246,0.2403,0.2454\right\},\left\{0.1765,0.2180,0.2153\right\},\\ \left\{0.2289,0.2674,0.2398\},\{0.2338,0.2328,0.1953\}\right\} \end{array}$$

**Figure 2:**
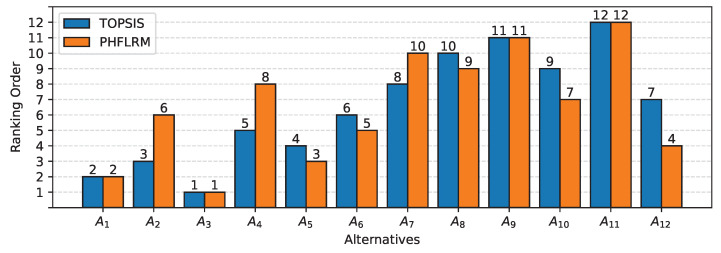
Ranking with PHFLRM and TOPSIS.

Further, the values of Euclidean distances ($\;D^{+}$ and $D^{-}$) and relative closeness ($P_{i}$) for each alternative are computed in Table 5 by using step 5, 6, and 7 of the algorithm (Section 4.1), as follows:

Table 5 shows that the best choice among the alternatives is $A_{3}$ as it has the largest value of $P_{i}$, whereas the alternative $A_{1}1$ is considered the worst choice of alternative, as it has the largest value of $P_{i}$. Further, two sets of ranking $R_{HFLLR}$ and $R_{TOPSIS}$ are compared using the bar chart in Figure 2, as follows:

Figure 2 illustrates that the ranking order between two sets of rankings, $R_{PHFLLR}$ and $R_{TOPSIS},$ is nearly similar, and that there is no significant difference between them. Although the graphical presentation provides a quick assessment of the performance of two ranking sets, $R_{PHFLLR}$ and $R_{TOPSIS}$, it is not conclusive. In order to determine the statistical significance of the two sets of rankings, the Spearman’s rank correlation coefficient is calculated, as shown in Table 6.

From Table 6, Spearman’s correlation coefficient is calculated as $r_{s}=1-\frac{6(38)}{1584}=0.87$, which shows that two sets of rankings, $R_{PHFLLR}$ and $\;R_{TOPSIS}$, are strongly related to each other [45]. To evaluate whether the correlation coefficient $r_{s}=0.87$ is meaningful or not, a statistical test is performed, taking the null hypothesis ($H_{0}$: there is no relationship between the two sets of rankings) against the alternative hypothesis ($H_{1}$: there is a relationship between two sets of rankings) at the 5% level of significance. As the calculated value, $Z_{c}$$r_{s}\sqrt{M-1}=0.87\sqrt{12-1}=2.88$, exceeds the table value $Z_{0.05}=1.645$, we reject $H_{0}$ and conclude that there is a very strong relationship between the two sets of rankings. Additionally, the values of correlation $r_{w}$ and similarity coefficient WS [46] were examined for the considered example. These values are 0.8607 and 0.9289, respectively, confirming the close correlation between the obtained results.

## 6. Conclusions

This paper provides a MCDM approach to FLRMs by incorporating probabilistic hesitant information. This concept has not been explored previously, and is a novel alternative to statistical regression in resolving MCDM challenges. The proposed methodology PHFLRM is applied in agriculture to evaluate wheat production in different Pakistan districts by considering significant factors such as rainfall, farm size, and irrigated area. We examined twelve districts’ yields across the country in the context of four factors that significantly affect wheat yield production. Similarly, we may include more criteria and alternatives, but computing becomes more complicated as the number of alternatives or criteria examined increases. Finally, the suggested methodology’s (PHFRM) outcomes are compared to the widely used decision-making technique called TOPSIS.

Compared with TOPSIS, the complexity of the proposed methodologies does not increase by inserting more criteria and alternatives into the given MCDM problems. The proposed methodology provides results by solving a simple LP model to obtain the ranking for decision-making problems, which provides results quickly, with less computational time than TOPSIS. The proposed methodologies may be a feasible alternative decision-making approach that accommodates a high-level system fuzziness. In the future, we will further investigate the applications of FLRM in decision-making using different FS extensions, and we should also investigate the accuracy of the obtained results.

## Figures and Tables

**Table 1:** Decision matrix H.

$A_{i}$	$Y_{i}$	$\;X_{i1}$	$X_{i2}$	$X_{i3}$
$A_{1}$	{475(0.35),478(0.30),482(0.35)}	{280(0.35),281(0.30),282(0.35)}	{23(0.25),24(0.30),25(0.35)}	{13.40(0.40),13.50(0.30)}
$A_{2}$	{520(0.35),524(0.35),530(0.30)}	{245(0.30),246(0.35),247(0.35)}	{25(0.35),26(0.30),27(0.25)}	{13.30(0.35),13.50(0.30),13.60(0.35)}
$A_{3}$	{436(0.35),438(0.35),439(0.30)}	{320(0.35),321(0.35),322(0.30)}	{24(0.25),25(0.30),26(0.25)}	{12.55(0.35),12.65(0.35),12.70(0.30)}
$A_{4}$	{530(0.35),536(0.30),540(0.30)}	{271(0.35),272(0.30),273(0.35)}	{25(0.40),26(0.30),30(0.30)}	{13.75(0.30),13.25(0.30),13.30(0.35)}
$A_{5}$	{496(0.40),500(0.30),506(0.30)}	{296(0.35),297(0.30),298(0.30)}	{26(0.35),27(0.35),27(0.30)}	{13.68(0.40),13.75(0.30),13.80(0.30)}
$A_{6}$	{520(0.40),526(0.30),530(0.30)}	{283(0.35),284(0.35),285(0.30)}	{27(0.35),28(0.30),29(0.30)}	{14.00(0.35),14.50(0.30),14.80(0.35)}
$A_{7}$	{540(0.35),545(0.35),551(0.30)}	{295(0.35),296(0.30),297(0.35)}	{28(0.35),28(0.30),30(0.30}	{14.18(0.35),14.25(0.35),14.30(0.30)}
$A_{8}$	{530(0.40),545(0.40),552(0.40)}	{269(0.30),270(0.35),271(0.35)}	{34(0.35),34(0.35),35(0.30)}	{14.23(0.40),14.35(0.30),14.40(0.30)}
$A_{9}$	{545(0.30),548(0.35),550(0.35)}	{243(0.35),250(0.30),255(0.30)}	{39(0.35),40(0.30)}	{13.30(0.40),13.50(0.30),13.60(0.30)}
$A_{10}$	{532(0.40),537(0.40),540(0.20)}	{303(0.30),304(0.35),305(0.30)}	{33(0.30),34(0.35),35(0.30)}	{13.38(0.30),13.45(0.35),13.65(0.35)}
$A_{11}$	{544(0.35),550(0.30),553(0.35)}	{303(0.30),304(0.35),305(0.35)}	{38(0.35),38(0.30),40(0.25)}	{13.35(0.30),13.45(0.30),13.55(0.40)}
$A_{12}$	{503(0.30),507(0.35),508(0.35)}	{250(0.30),251(0.35),252(0.30)}	{31(0.30),32(0.35),33(0.35)}	{12.63(0.30),13.75(0.35),}

**Table 2:** Decision matrix.

$A_{i}$	$Y_{i}$	$\;X_{i1}$	$X_{i2}$	$X_{i3}$
$A_{1}$	{475(0.35),478(0.30),482(0.35)}	{280(0.35),281(0.30),282(0.35)}	{23(0.25),24(0.30),25(0.35)}	{13.40(0.40),13.50(0.30),13.50(0.30)}
$A_{2}$	{520(0.35),524(0.35),530(0.30)}	{245(0.30),246(0.35),247(0.35)}	{25(0.35),26(0.30),27(0.25)}	{13.30(0.35),13.50(0.30),13.60(0.35)}
$A_{3}$	{436(0.35),438(0.35),439(0.30)}	{320(0.35),321(0.35),322(0.30)}	{24(0.25),25(0.30),26(0.25)}	{12.55(0.35),12.65(0.35),12.70(0.30)}
$A_{4}$	{530(0.35),536(0.30),540(0.30)}	{271(0.35),272(0.30),273(0.35)}	{25(0.40),26(0.30),30(0.30)}	{13.75(0.30),13.25(0.30),13.30(0.35)}
$A_{5}$	{496(0.40),500(0.30),506(0.30)}	{296(0.35),297(0.30),298(0.30)}	{26(0.35),27(0.35),27(0.30)}	{13.68(0.40),13.75(0.30),13.80(0.30)}
$A_{6}$	{520(0.40),526(0.30),530(0.30)}	{283(0.35),284(0.35),285(0.30)}	{27(0.35),28(0.30),29(0.30)}	{14.00(0.35),14.50(0.30),14.80(0.35)}
$A_{7}$	{540(0.35),545(0.35),551(0.30)}	{295(0.35),296(0.30),297(0.35)}	{28(0.35),28(0.30),30(0.30}	{14.18(0.35),14.25(0.35),14.30(0.30)}
$A_{8}$	{530(0.40),545(0.40),552(0.40)}	{269(0.30),270(0.35),271(0.35)}	{34(0.35),34(0.35),35(0.30)}	{14.23(0.40),14.35(0.30),14.40(0.30)}
$A_{9}$	{545(0.30),548(0.35),550(0.35)}	{243(0.35),250(0.30),255(0.30)}	{39(0.35),40(0.30),40(0.30)}	{13.30(0.40),13.50(0.30),13.60(0.30)}
$A_{10}$	{532(0.40),537(0.40),540(0.20)}	{303(0.30),304(0.35),305(0.30)}	{33(0.30),34(0.35),35(0.30)}	{13.38(0.30),13.45(0.35),13.65(0.35)}
$A_{11}$	{544(0.35),550(0.30),553(0.35)}	{303(0.30),304(0.35),305(0.35)}	{38(0.35),38(0.30),40(0.25)}	{13.35(0.30),13.45(0.30),13.55(0.40)}
$A_{12}$	{503(0.30),507(0.35),508(0.35)}	{250(0.30),251(0.35),252(0.30)}	{31(0.30),32(0.35),33(0.35)}	{12.63(0.30),13.75(0.35),13.75(0.35)}

**Table 3:** Estimated values obtained by PHFLRM.

k=1	k=2	k=3
$\lambda_{1}^{1}=1.0000$	$\lambda_{1}^{2}=1.0000$	$\lambda_{1}^{3}=1.0000$
$\lambda_{2}^{1}=1.0000$	$\lambda_{2}^{2}=0.9872$	$\lambda_{2}^{3}=1.0000$
$\lambda_{3}^{1}=1.0000$	$\lambda_{3}^{2}=1.0000$	$\lambda_{3}^{3}=0.9975$
$\lambda_{4}^{1}=1.0000$	$\lambda_{4}^{2}=1.0000$	$\lambda_{4}^{3}=1.0000$
$\lambda_{5}^{1}=1.0000$	$\lambda_{5}^{2}=1.0000$	$\lambda_{5}^{2}=1.0000$
$\lambda_{6}^{1}=1.0000$	$\lambda_{6}^{2}=1.0000$	$\lambda_{6}^{2}=1.0000$
$\lambda_{7}^{1}=1.0000$	$\lambda_{7}^{2}=1.0000$	$\lambda_{7}^{2}=1.0000$
$\lambda_{8}^{1}=1.0000$	$\lambda_{8}^{2}=1.0000$	$\lambda_{8}^{3}=1.0000$
$\lambda_{9}^{1}=0.9824$	$\lambda_{9}^{2}=1.0000$	$\lambda_{9}^{3}=1.0000$
$\lambda_{10}^{1}=1.0000$	$\lambda_{10}^{2}=1.0000$	$\lambda_{10}^{3}=0.9797$
$\lambda_{11}^{1}=1.0000$	$\lambda_{11}^{2}=1.0000$	$\lambda_{11}^{3}=1.0000$
$\lambda_{12}^{1}=1.0000$	$\lambda_{12}^{2}=1.0000$	$\lambda_{12}^{3}=1.0000$
$\alpha_{0}^{1}=0.5984$	$\alpha_{0}^{2}=0.8602$	$\alpha_{0}^{3}=0.4812$
$\alpha_{1}^{1}=2.1288$	$\alpha_{1}^{2}=15.5364$	$\alpha_{1}^{3}=8.8699$
$\alpha_{2}^{1}=3.2602$	$\alpha_{2}^{2}=11.1963$	$\alpha_{2}^{3}=1.6489$
$\alpha_{3}^{1}=0.3059$	$\alpha_{3}^{2}=-1.0018$	$\alpha_{3}^{3}=0.2233$
$c_{0}^{1}=0.0000$	$c_{0}^{2}=0.03757$	$c_{0}^{3}=0.0000$
$c_{1}^{1}=0.0000$	$c_{1}^{2}=0.0000$	$c_{1}^{3}=1.4431$
$c_{2}^{1}=1.3864$	$c_{2}^{2}=1.7105$	$c_{2}^{3}=4.7510$
$c_{3}^{1}=0.1913$	$c_{3}^{2}=0.0000$	$c_{3}^{3}=0.0000$

**Table 4:** Ranking with PHFLRM ($R_{PHFLR}$).

$A_{i}$	$\mathrm{Sc}(Y_{i})$	$\mathrm{Sc}(Y_{i}^{*})$	$e_{i}$	$R_{\mathrm{PHFLR}}$
$A_{{}_{1}}$	37.0085	0.7223	36.2862	2
$A_{{}_{2}}$	44.2253	0.7203	43.5050	6
$A_{{}_{3}}$	30.7738	0.7255	30.04823	1
$A_{{}_{4}}$	46.0855	0.7258	45.3597	8
$A_{{}_{5}}$	40.1481	0.7305	39.4176	3
$A_{{}_{6}}$	44.1899	0.7333	43.4566	5
$A_{{}_{7}}$	47.7795	0.7362	47.0433	10
$A_{{}_{8}}$	46.3455	0.7465	45.5989	9
$A_{{}_{9}}$	48.6282	0.7526	47.8756	11
$A_{{}_{10}}$	45.4646	0.7476	44.7179	7
$A_{{}_{11}}$	48.7416	0.7578	47.9838	12
$A_{{}_{12}}$	41.5150	0.7352	40.7798	4

**Table 5:** Ranking using the TOPSIS (Section 4.1) approach.

$A_{i}$	$D^{+}$	$D^{-}$	$P_{i}$	$R_{\mathrm{TOPSIS}}$
$A_{1}$	0.3595	0.1877	0.6570	2
$A_{2}$	0.3311	0.1781	0.6503	3
$A_{3}$	0.3585	0.1850	0.6591	1
$A_{4}$	0.2976	0.2094	0.5870	5
$A_{5}$	0.3075	0.2156	0.5878	4
$A_{6}$	0.2764	0.2262	0.5500	6
$A_{7}$	0.2497	0.2465	0.5032	8
$A_{8}$	0.2446	0.2948	0.4535	10
$A_{9}$	0.2051	0.3605	0.3627	11
$A_{10}$	0.2478	0.2936	0.4577	9
$A_{11}$	0.1856	0.3731	0.3323	12
$A_{12}$	0.2817	0.2385	0.5416	7

**Table 6:** Spearman’s rank correlation coefficients.

$A_{i}$	$R_{\mathrm{PHFLLR}}$	$R_{\mathrm{TOPSIS}}$	$d^{2}$
$A_{1}$	2	2	0
$A_{2}$	3	6	9
$A_{3}$	1	1	0
$A_{4}$	5	8	9
$A_{5}$	4	3	1
$A_{6}$	6	5	1
$A_{7}$	8	10	4
$A_{8}$	10	9	1
$A_{9}$	11	11	0
$A_{10}$	9	7	4
$A_{11}$	12	12	0
$A_{12}$	7	4	9

## Data Availability

Not applicable.

## Abbreviations

The following abbreviations are used in this manuscript:

MCDM	Multi-Criteria Decision-Making;
HFS	Hesitant Fuzzy Set;
PHFS	Probabilistic Hesitant Fuzzy Set;
FLRM	Fuzzy Linear Regression Model;
PHFE	Probabilistic Hesitant Fuzzy Elements;
IPOP	Input–Output;
LPM	Linear Programming Model;
PHFLRM	Probabilistic Hesitant Fuzzy Linear Regression Model;
TOPSIS	Technique For Order Preference by Similarity to Ideal Solution.
